# Effects of Oxalis barrelieri L. (Oxalidaceae) aqueous extract on diarrhea induced by *Shigella dysenteriae* type 1 in rats

**DOI:** 10.1002/hsr2.20

**Published:** 2017-12-07

**Authors:** Michel Archange Fokam Tagne, Paul Aimé Noubissi, Gaëtan Olivier Fankem, René Kamgang

**Affiliations:** ^1^ Department of Biological Science, Faculty of Science University of Ngaoundéré Ngaoundéré Cameroon; ^2^ Animal Physiology Laboratory, Faculty of Science University of Yaoundé I Yaoundé Cameroon; ^3^ Department of Zoology and Animal Physiology, Faculty of Science University of Buéa Buea Cameroon; ^4^ Endocrinology and Radioisotopes Laboratory Institute of Medical Research and Medicinal Plants Studies (IMPM) Yaoundé Cameroon

**Keywords:** antibacterial susceptibility, antidiarrheal activity, Oxalis barrelieri, rat, *Shigella dysenteriae* type 1

## Abstract

**Aim:**

Oxalis barrelieri is a medicinal plant commonly used in Cameroon, for the treatment of many diarrheal diseases. The antibacterial properties of O barrelieri aqueous extract (WOb) against Shigella dysenteriae type 1 were investigated in vitro and in vivo.

**Methods:**

**A**ntibacterial activity was evaluated in vitro by disc diffusion method and by macrodilution method. S dysenteriae type 1 at a dose of 1.2 × 10^9^ CFU was administrated orally to rats to induce shigellosis. For 6 consecutive days, diarrheic rats were treated with O barrelieri aqueous extract (50 and 100 mg/kg BW) or norfloxacin (20 mg/kg BW). The diarrheal stool weight and S dysenteriae type 1 density were assessed during the treatment period, and death rate recorded. Nitric oxide production in blood and in colonic homogenate and blood parameters were assessed, and the histological section of the colon was performed in the survivors.

**Results:**

The minimal inhibitory concentration and minimal bactericidal concentration of WOb were, respectively, 6 mg/mL and 25 mg/mL. The mean minimal bactericidal concentration/minimal inhibitory concentration ratio for WOb against S dysenteriae type 1 was high (˃4); WOb could be classified as a bacteriostatic drug. WOb significantly (P < .01) reduced bacterial density and diarrheal stool weight. WOb decreased nitric oxide production (P < .01) in the large intestine and protected the mucosa of the colon from bacterial destruction.

**Conclusion:**

The results suggest that O barrelieri aqueous extract possesses bacteriostatic and antidiarrheal activities and reduces damages caused to intestinal mucosa barrier by pathogenic mechanisms of Shigella. This extract could be used as an alternative therapeutic for infectious diarrhea.

## Abstract 2

Aqueous extract of *Oxalis barrelieri* contains chemical compounds, which have antibacterial properties in vitro and in vivo on Shigella strains. This extract is bacteriostatic, protects the intestinal mucosa against damage caused by *Shigella dysenteriae* type I, and maintains the normal immune system. This extract could be used as an alternative therapeutic for infectious diarrhea.

## 1 INTRODUCTION

In mammals, the gastrointestinal tract harbors various microbes that play an essential role in maintaining its physiological homeostasis.^1^ Changes in the composition of these gut microbes can alter the intestinal barrier and the immune system,^2^, ^3^ which can lead to gastrointestinal infections. Gastrointestinal infections caused by bacteria, viruses, and parasites are usually manifested by diarrhea or by inflammatory bowel diseases or gastroenteritis.^4^ Diarrhea is defined as the emission of at least 3 nonmolded or liquid stools per day.^5^, ^6^ It is an alteration in normal bowel movement that leads to the increase in water and electrolyte content, volume or liquid stool frequency, and abdominal pain.^7^ An emission of 10 g/kg BW/d of feces in infants and children and 200 g/kg BW/d in an adolescent or an adult is considered abnormal.^8^ Diarrhea can be infectious or not. Noninfectious diarrhea can be due to hormones that accelerated intestine transit, to osmotic substances and/or laxatives. Infectious diarrhea can be due to virus, bacteria, and/or parasites.^4^ In the world and particularly in developing countries, infectious diarrhea still remains one of the leading causes of infant mortality.^9^ A multicentric study from 6 Asian countries estimated *Shigella* as the causative agent in 5% of the diarrheal cases.^10^ As little as 10 to 100 *Shigella* can cause shigellosis in human,^11^ whereas 1.2 × 10^9^ *Shigella* can cause dysenteric diarrhea in rats.^12^ Shigellosis occurs worldwide, in sporadic, endemic, epidemic, and pandemic forms.^13^ Most of the cases are children <5 years of age. The annual number of shigellosis episodes throughout the world is estimated to be 164.7 million, with 69% of all episodes and 61% of all deaths attributable to shigellosis involving children <5 years of age.^10^ The economic impact of diarrhea and its treatment are of considerable importance.^14^, ^15^ The annual treatment costs for diarrhea are very high and ranged from US$ 907 116 to US$ 1 851 280 for ambulatory clinical consultations and from US$ 701 833 to US$ 4 581 213 for hospitalizations.^16^ The re‐emergence of *Shigella dysenteriae* type 1 (*Sd1*) with added resistance to ciprofloxacin, which has epidemic potential, has also been reported.^10^ Many synthetic drugs such as diphenoxylate and loperamide are available for the treatment of diarrhea, but they have toxic side effects.^17^ Therefore, the search for new, safe, more effective, and less toxic molecules has continued to be an important area of research in pharmacology. Since antiquity, diarrhea has been treated with medicinal plants in traditional medicine.^17^ Oxalis barrelieri L. is traditionally used by Cameroon inhabitants (Central Africa) for diarrhea care. The O barrelieri aqueous extract, at doses of 50 and 100 mg/kg BW, showed significant antidiarrheal activities in rats treated with castor oil.^18^ Phytochemical studies of O barrelieri aqueous extract revealed the presence of compounds such as phenols, terpenoids, anthocyanidins, anthraquinones, coumarins, and saponins.^18^ This work has been initiated to evaluate the antidiarrheal properties of O barrelieri aqueous extract on infectious diarrhea, by using *Sd1*–induced diarrhea in rats as a model.

## 2 MATERIALS AND METHODS

### 2.1 Plant material

Whole plants of O barrelieri were collected in Yaoundé (Center Region of Cameroon) in September 2014 between 8 AM and 10 AM. The plant was identified at the National Herbarium of Cameroon in Yaoundé, and a voucher specimen was deposited under no. 49998 HNC. Whole plants were washed thoroughly with water, shade‐dried, and ground. The powder (407 g) was macerated in distilled water (5 L) for 3 days in a percolator. After 3 days of maceration, the mixture was filtered by opening the tap of the percolator and was concentrated by vacuum distillation at 50°C to yield 128 g (31.45%) of brown extract. A stock solution (10 mg/mL) was prepared by dissolving 10 g of dry extract in distilled water to obtain 1000 mL of solution.

### 2.2 Experimental animals

Prior to the study, male and female Wistar albino rats (60‐98 g), approximately 6 weeks old, were selected and allowed to acclimatize for 1 week to our laboratory environment (22°C‐25°C and 12 h light/12 h of darkness). In vivo experiments on rats were performed according to the European Union guidelines on animal care (CEE Council 86/609) that was adopted by the Ministry of Scientific Research and Innovation of Cameroon.^19^ Animals housed in metabolic cages (1 animal/cage) were fed a diet consisting of carbohydrates (50%‐55%), fats (15%‐20%), and proteins (25%‐30%).^20^

### 2.3 Bacterial strain

Clinical isolates of *Sd1* from patients with severe infections were provided by the Centre Pasteur of Yaoundé, Cameroon.

### 2.4 In vitro antibacterial susceptibility

#### 2.4.1 Disc diffusion method

**Figure 1:**
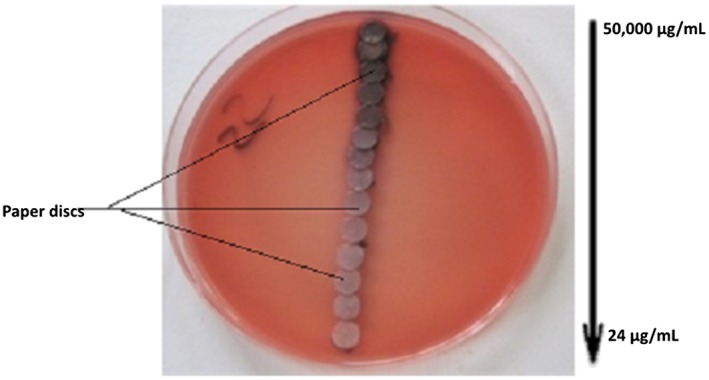
Realization of the adapted E‐test method with the *Oxalis barrelieri* aqueous extract on *Salmonella Shigella* agar inoculated with *Shigella dysenteriae* type 1

Minimal inhibitory concentration (MIC) was determined with adapted E‐test according to disc diffusion method.^21^ Sterile 6‐mm Ø filter paper discs (Schleicher & Schul, no. 2668, Dassel, Germany) were impregnated with 50 μL of O barrelieri aqueous extract at various concentrations. *Sd1* inoculum of 0.5 mL (5 × 10^5^ CFU) was flooded in *Salmonella Shigella* (SS) and in Mueller Hinton agar (Oxoid) and incubated for 15 minutes at 37°C. After incubation, the impregnated paper discs were placed on the plates according to decreasing extract concentrations (50 000‐24 μg/mL) (Figure 1). The petri dishes were incubated at 37°C for 24 hours. After 24‐hour incubation at 37°C, the growth inhibition and the MIC (first disc that did not present growth inhibition) were observed on the petri dishes. The test was performed under sterile conditions in duplicate and repeated 3 independent times.

#### 2.4.2 Macrodilution method

This method has been adopted from NCCLS M26‐A,^22^ with modifications.^23^, ^24^ The *Sd1* strains were adjusted to achieve a turbidity equivalent to a 0.5 McFarland (1 × 10^8^ CFU/mL) and diluted (1:1000)^25^ in brain heart infusion (Oxoid). A dilution series of the extract, ranging from 50 000 to 24 μg/mL, were prepared and then transferred to the broth in 14 tubes. A 1.0‐mL extract was pipetted into tubes by twofold dilutions. Freshly grown bacteria of 1.0 mL were added to the tubes in a density of 10^5^ CFU/mL (final concentration/tube). The tubes were incubated overnight at 37°C. CFU was determined by diluting each well in tenfold dilutions. From each dilution, aliquots were transferred to agar plates and incubated overnight. On the following day, the number of colonies was evaluated, and the initial CFU/tube retrospectively calculated by the formula:

$$\text{Number of}\;\mathrm{Sd}1\left(\mathrm{CFU}\right)=\frac{\text{number of colonies}}{\text{Volume of dilution}\;x\;\text{Dilution factor}}.$$

26

The lowest concentrations of extract that did not show any visible growth after macroscopic evaluation were considered to be the MIC.^27^ Minimal bactericidal concentration (MBC, concentration producing 99.99% reduction of CFU [10^3^ CFU/mL] in the initial inoculum) was determined by subculture on nutrient agar. For nutrient agar subculture, culture broths that did not show visible bacterial growth (no turbidity) were seeded on Mueller Hinton agar and SS agar for 24 hours at 37°C. Minimal bactericidal concentration was determined as the lowest concentration of extract that did not show bacterial growth in subcultures.^28^ Antibacterial activity was determined by the MBC/MIC formula.^29^

### 2.5 *Sd1*‐induce diarrhea

#### 2.5.1 Diarrhea induction and treatment

Rats were housed separately in metabolic cages. Before diarrhea induction, we checked that our animals were not carrying *Shigella*. In normal animals, stools were removed by rectal curettage using a tongue depressor. A 0.5‐g stool was dissolved in 4.5 mL of sterile saline solution, and 0.5 mL of the solution was inoculated on SS agar plate and incubated for 24 hours at 37°C. The animals from which the stool cultures were positive were excluded. After verifying that the rats were not carrying *Sd1*, diarrhea was induced by orally administering to each rat a solution of 1.2 × 10^9^ saline‐diluted *Sd1* cells.^12^, ^30^, ^31^, ^32^

When diarrhea appeared (26 h after administration of *Shigella* inoculum), the rats were randomly divided into 6 groups of 5 animals each. Groups 1 and 2, diarrheic control (DC), did not receive any treatment, but Group 1 was sacrificed 2 days after induction to determine haematological parameters and nitric oxide (NO) level. Three other groups were treated with antidiarrheal drugs twice daily (6:00 AM and 6:00 PM) for 6 consecutive days: Group 3 (Nor 20), 4 (WOb50), and 5 (WOb100) received, respectively, 20 mg/kg BW antibiotic norfloxacin (positive control, norfloxacine: A‐320 norfen 400 mg tablet; Cadila Pharmaceuticals Ltd), 50 mg/kg BW, and 100 mg/kg BW aqueous extract of O barrelieri. To treat animals with the 100 mg/kg dose, we administered orally stock solution (10 mg/mL) at 10 mL/kg BW to each animal. For the 50 mg/kg BW dose, the stock solution was diluted twice and then administered orally to the animals at 10 mL/kg BW solution. To exclude food involvement in induction of diarrhea, Group 6 (normal control [NC]) consisting of 5 normal rats received food and water, but no bacterial inoculums or drug. This group was treated only with distilled water (10 mL/kg BW) for 6 consecutive days.

The number of deaths was recorded during treatment. The stools were collected daily using a sterile stool pot of the metabolic cage. The weight and quality of feces were examined daily for 6 consecutive days of treatment. *Sd1* counts in feces were done before induction of diarrhea and daily for 6 consecutive days after onset of diarrhea. For this purpose, 0.5 g of stool was dissolved in 4.5 mL of sterile saline solution, serial dilutions were made, and 0.5 mL of each dilution was inoculated on the SS agar plate and incubated for 24 hours at 37°C.^30^ After incubation, the number of *Sd1* was determined.^33^ After 6 days of treatment, all survival animals were sacrificed and their blood and their colon were collected for blood cells count using manual method^34^ and/or for NO test using the modified Griess method.^35^ Colon fragments were fixed in 10% buffered formalin for histopathological examination.^30^

### 2.6 NO dosage

Nitric oxide concentration was evaluated in serum and in colon homogenates.^30^ To obtain Griess solution, 0.25 mL of Griess 1 (0.8 g sulfanilic acid + 250 mL acetic acid 30%) was added to 0.25 mL of Griess 2 (0.05 g of α‐naphthylamine + 100 mL acetic acid 30%). A 0.5‐mL serum or homogenate of the colon was added to 0.5 mL of Griess solution, and the mixture obtained was left for 20 minutes at room temperature. After 20 minutes, the optical density of each mixture was read using a spectrophotometer (T60‐1611ESW) at 553 nm and recorded.^35^

### 2.7 Hematological studies

Figured elements of the blood (red blood cells [RBC], white blood cells [WBC], and platelet cells [PC]) were counted by a manual method^34^ using a light microscope (MOTIC 1820 LED: SM7432‐MC1ST‐RPIWFM). The hematocrit (Ht) of each rat was determined using the microhematocrit tube. Hemoglobin level (Hb) was determined by the spectrophotometric method. The blood was diluted in a Drabking solution (1/250), and the absorbance was read at 510 nm and recorded. Mean corpuscular volume (MCV), mean corpuscular hemoglobin (MCH), and MCH concentration (MCHC) were calculated, respectively, by the formulae:

$$\mathrm{MCV}=\frac{\mathrm{Ht}}{\mathrm{RBC}}\;x\;10;$$

36

$$\mathrm{MCH}=\frac{\mathrm{Hb}\;}{\mathrm{RBC}}\;x\;10;$$

37

$$\text{MCHC}=\frac{\mathrm{Hb}}{\mathrm{Ht}}\;x\;10.$$

36

### 2.8 Histopathological investigations

Histopathological investigations were done according to methods described in the literature.^38^ For this purpose, colon fragments of each experimental rat were fixed in 10% buffered formalin in labeled flasks for histological examination. These colon fragments were embedded in paraffin wax, and 2‐μm‐thick sections were made with the microtome. These preparations were mounted on glass slides that were then stained with hematoxylin and eosin and examined under a standard light microscope (MOTIC 1820 LED: SM7432‐MC1ST‐RPIWFM).^39^

### 2.9 Statistical analysis

The data are means ± standard error of the mean ( $\bar{X\;}$ ± SEM) expressed in tables, figures, and photos. These data were analyzed by 1‐way analysis of variance followed by the Dunnett *t* test and the Tukey test using Computer Pad InStat 3.05 (Graph Pad software, USU).

## 3 RESULTS

### 3.1 Susceptibility of *Sd1* to O barrelieri aqueous extract

**Figure 2:**
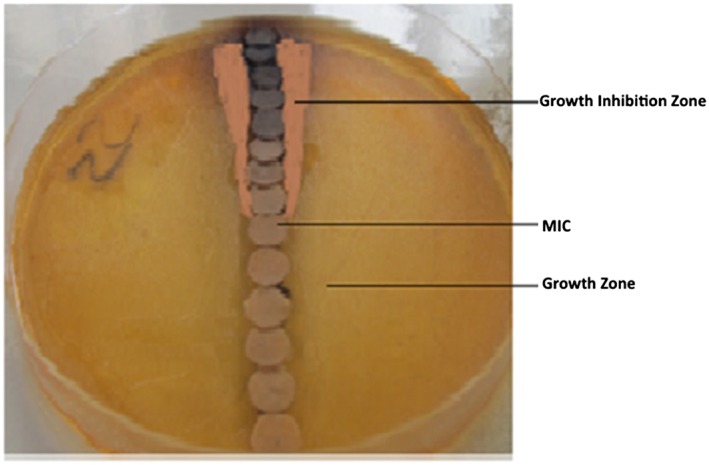
Sensitivity of *Shigella dysenteriae* type 1 to the *Oxalis barrelieri* aqueous extract on *Salmonella Shigella* agar medium. MIC, minimal inhibitory concentration

In vitro, the O barrelieri water extract showed an intersection point between the growth zone and the growth inhibition zone corresponding to the MIC. This MIC was about 6 mg/mL (Figure 2).

### 3.2 MIC and MBC values of O barrelieri water extract by the graphic method

**Figure 3:**
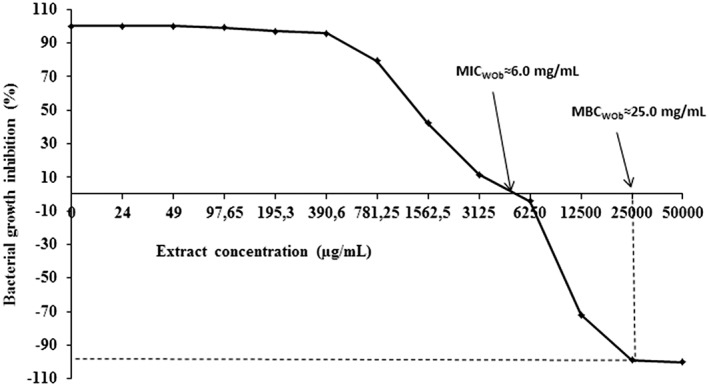
Bacterial growth inhibition (%) of *Oxalis barrelieri* aqueous extract (WOb) in vitro. MBC, minimal bactericidal concentration; MIC, minimal inhibitory concentration

The O barrelieri water extract (WOb) showed in vitro inhibitory activity on *Shigella* growth. The MIC and MBC values were 6 mg/mL and 25 mg/mL, respectively (Figure 3). The MBC/MIC ratio was 4.16.

### 3.3 Antidiarrheal activities

**Figure 4:**
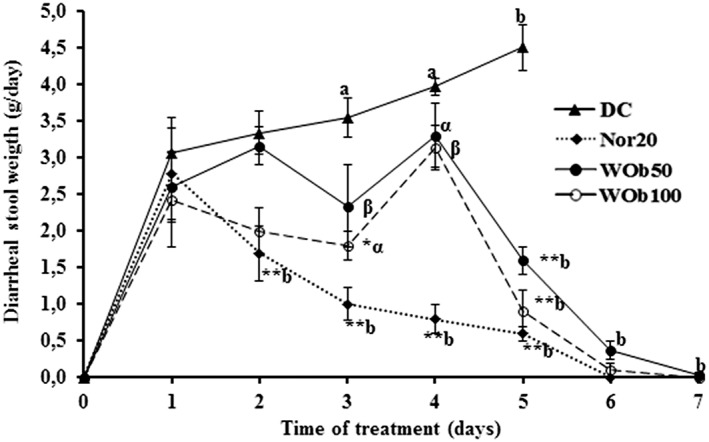
Diarrheal stool weight during the treatment of diarrheic rats with *Oxalis barrelieri* aqueous extract 50 mg/kg BW (WOb50), 100 mg/kg BW (WOb100), and norfloxacin 20 mg/kg BW (Nor20). Data are the means ± SEM (n = 5). Significant difference: *P < .05 and **P < .01 compared with diarrheic control (DC) rats; ^a^ *P* < .05 and ^b^ *P* < .01 compared with initial values (d1: diarrhea appearance and treatment start); ^α^ *P* < .05 and ^β^ *P* < .01 compared with reference drug (Nor20). d0: Sd1 administration

Normal rats that were not inoculated with *Shigella* and were treated with distilled water showed no signs of diarrhea. A few hours (4 h) after *Sd1* inoculum administration, the rats became calm, less mobile, curled up, and showed erect hairs. Twenty‐six hours after inoculum administration, the rats emitted the first diarrheal stool and became more aggressive. During the treatment, rats recovered mobility progressively, and their aggressiveness decreased. Diarrheal stools were soft or liquid containing mucus or blood marks and attracted flies by their fetid odors. These symptoms disappeared after 3 days of treatment. During treatment, no death was recorded in all treated groups and NC. However, we recorded 100% death in DC group (Table 1). The first day of onset of diarrhea, stool weights were 3.06 ± 0.49 g, 2.78 ± 0.62 g, 2.60 ± 0.37 g, and 2.42 ± 0.37 g, respectively, for TD, Nor20, WOb50, and WOb100. These values increased in untreated group over time, decreased significantly (*P* < .01) from the second day in norfloxacin‐treated group (Nor20) and from the third day in extract‐treated groups (Figure 4). Diarrhea was eliminated by the sixth day of treatment.

**Table 1:** Mortality rate (%) of diarrheic rats during 6 days of treatment (n = 5)

Day Treatment	Diarrheic Control	Norfloxaxine (20 mg/kg)	Aqueous Extract of Oxalis barrelieri	
			50 mg/kg	100 mg/kg
1	0	0	0	0
2	0	0	0	0
3	40	0	0	0
4	60	0	0	0
5	100	0	0	0
6	…	0	0	0

**Figure 5:**
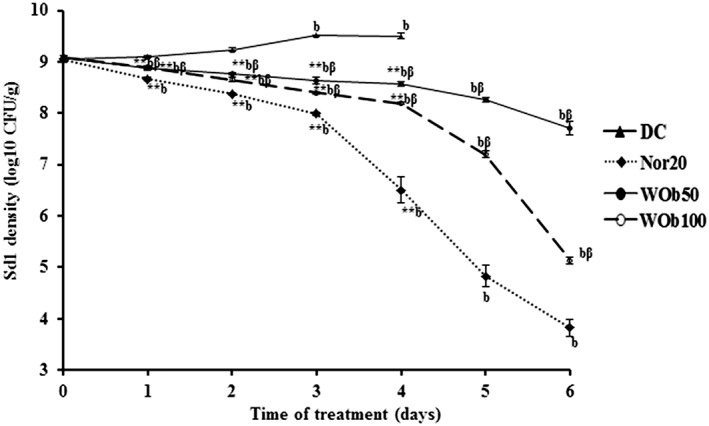
*Shigella dysenteriae* A1 density (log10 transformed) in diarrheic rat stools over 6 days treatment with 50 mg/kg BW (WOb50) and 100 mg/kg BW (WOb100) *Oxalis barrelieri* water extract and norfloxacin 20 mg/kg BW (Nor20). Data are the mean ± SEM (n = 5). Significant difference: *P < .05 and **P < .01 compared with diarrheic control (DC) rats; ^a^ *P* < .05 and ^b^ *P* < .01 compared with initial values; ^β^ *P* < .01 compared with reference drug (Nor20). d1: diarrhea appearance and treatment start

After onset of diarrhea in rats, the number of *Sd1* was about 1.2 × 10^9^ in all groups. These values increased significantly (*P* < .05) from third day in untreated group. In all treated groups, these values decreased significantly (*P* < .01) to 0.9 × 10^4^, 59.4 × 10^6^, and 14.1 × 10^4^ CFU, respectively, for Nor20, WOb50, and WOb100 at the sixth day of treatment (Figure 5).

### 3.4 Effect of O barrelieri on NO production

**Figure 6:**
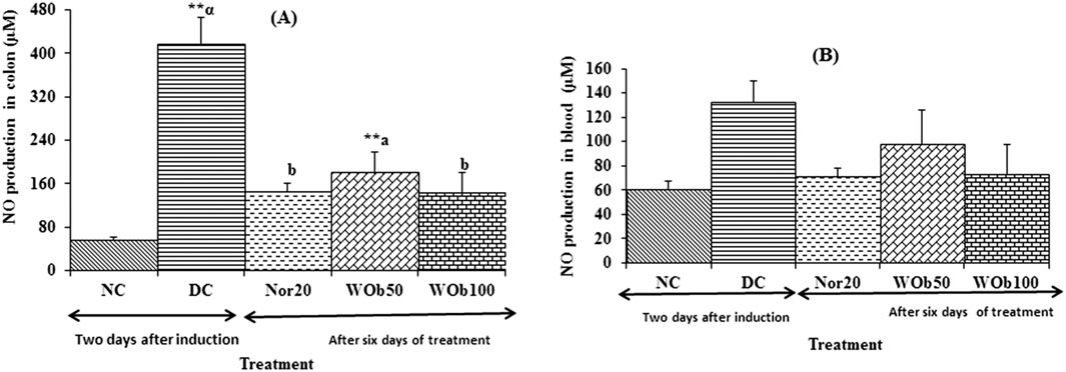
Nitric oxide (NO) production in colon (A) and blood (B) in diarrheic rats after 6 days treatment with norfloxacin 20 mg/kg BW (Nor20) and *Oxalis barrelieri* aqueous extract 50 mg/kg BW (WOb50) and 100 mg/kg BW (WOb100). Data are mean ± SEM (n = 5). Significant difference: **P < .01 compared with normal control (NC); ^a^ *P* < .05 and ^b^ *P* < .01 compared with diarrheic control (DC); ^α^ *P* < .01 compared with reference drug (Nor20)

In DC rats, NO production in colon was markedly high compared to NC: 418.15 ± 48.65μM against 57.34 ± 3.76μM (*P* < .01), 2 days after the onset of diarrhea. After 6 days of treatment, NO production in colon was significantly reduced (*P* < .01) by 20 mg/kg BW norfloxacin and by 50 and 100 mg/kg BW aqueous extract of O barrelieri after the sixth day of treatment (Figure 6A). The blood level of NO was higher in DC than normal rats: 132.42 ± 17.26μM against 60.59 ± 6.61μM. After 6 days of therapy, neither the norfloxacin nor the O barrelieri extract significantly reduced the production of NO in diarrheic rats blood (Figure 6B).

### 3.5 Blood parameters

Red blood cells, Ht, PC, MCV, MCH, and MCHC did not significantly change in norfloxacin‐treated diarrheic rats compared to NC. However, in O barrelieri water extract treated, only the WBC and hemoglobin decreased significantly (*P* < .01) compared to NC rats: 6.55 ± 0.38 × 10^3^/mm^3^ and 10.39 ± 0.39 × 10^3^/mm^3^ for WBC and 14.63 ± 0.67 g/dL and 17.65 ± 0.73 g/dL for hemoglobin, respectively (Table 2).

**Table 2:** Blood cells rate in diarrheic rats after 6 days treatment with *Oxalis barrelieri* aqueous extract 50 mg/kg BW (WOb50), 100 mg/kg BW (WOb100), and norfloxacin 20 mg/kg BW (Nor20)

Group	NC	DC	Nor 20 mg/kg	WOb 50 mg/kg	WOb 100 mg/kg
WBC × 10^3^/mm^3^	10.7 ± 0.5	6.5 ± 0.4**	10.4 ± 0.4^b^	6.8 ± 0.3**	5.4 ± 0.2**
Hb, g/dL	17.6 ± 0.7	14.6 ± 0.7*	16.2 ± 0.5	13.9 ± 0.7**	17.1 ± 0.2
Ht, %	49.6 ± 2.4	41.8 ± 2.8**	46.3 ± 1.9	40.5 ± 2.9**	51.0 ± 0.7
RBC × 10^6^/mm^3^	8.0 ± 0.3	7.1 ± 0.2*	7.6 ± 0.1	7.1 ± 0.3*	7.7 ± 0.3
PC × 10^6^/mm^3^	84.6 ± 5.4	68.1 ± 2.0*	80.9 ± 1.4	86.7 ± 3.3	86.2 ± 3.1
MCV, μm^3^	61.7 ± 1.2	58.9 ± 3.5	60.6 ± 2.6	57.0 ± 2.2	66.7 ± 2.1
MCH, ρg	21.9 ± 0.0	20.6 ± 0.8	21.3 ± 0.8	19.6 ± 0.7	22.3 ± 0.9
MCHC, ρg/dL	35.6 ± 0.8	35.2 ± 0.8	35.2 ± 0.5	34.5 ± 1.0	33.5 ± 0.4

Abbreviations: DC, diarrheic control; Hb, hemoglobin; MCH, mean corpuscular hemoglobin; MCHC, mean corpuscular hemoglobin concentration; MCV, mean corpuscular volume; NC, normal control; PC, platelet cells; RBC, red blood cells; WBC, white blood cells.

Data are the mean ± SEM (n = 5 per group). Significant difference: *P < .05. **P < .01 compared with NC. ^b^ *P* < .01 compared with DC.

### 3.6 Effects of O barrelieri water extract on the microhistology of colon in diarrheal rats

**Figure 7:**
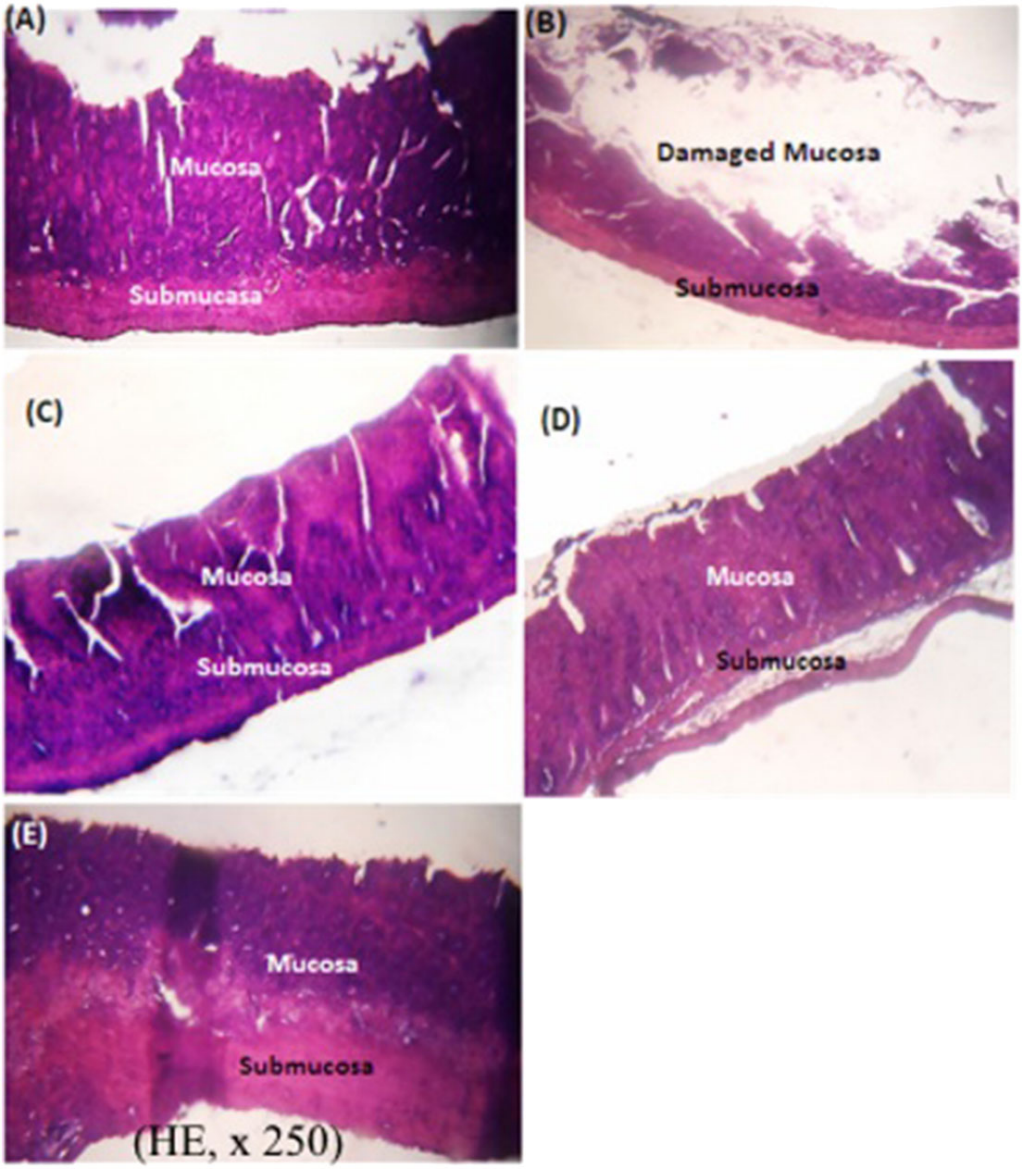
Histological section of the colon of normal rats (A), untreated diarrheal rats (B) and diarrheal rats treated with norfloxacin 20 mg/kg (C), and the *Oxalis barrelieri* water extract 50 mg/kg (D) or 100 mg/kg (E)

The control rat colon (Figure 7A) showed normal mucosa. In untreated diarrheal rats, colon histology showed destruction of the mucosa (Figure 7B). However, in rats treated with norfloxacin 20 mg/kg (Figure 7C), with 50 mg/kg (Figure 7D) or 100 mg/kg (Figure 7E) O barrelieri aqueous extract, the microhistology of the colon showed a normal mucosa.

## 4 DISCUSSION

The purpose of this study was to provide scientific support for the traditional use of O barrelieri extract to treat diarrhea. Antidiarrheic effects of O barrelieri aqueous extract were investigated using the antibacterial activity in vitro on *Sd1* and the activity on shigellosis induced in rats.

In vitro antimicrobial study showed an inhibitory activity of extract against *Sd1* growth. Minimal inhibitory concentration and MBC values of O barrelieri aqueous extract were high compared to norfloxacin MIC value (0.5 to >256 μg/L),^40^ although they are closer to those reported for other plant extracts, eg, 0.8 mg/mL with the methanol extract of *Picralima nitida*,^41^ 1.17 mg/mL with aqueous extract of *Mallotus oppositifolium*,^32^ and 3.5 mg/mL with aqueous ethanol extract of Euphorbia prostrata ait.^31^ These results are probably due to the fact that this crude extract might contain less active compounds against this bacterial strain. For aqueous extract of O barrelieri, the ratio MBC/MIC was higher than 4, and this could thus indicate a bacteriostatic activity.^29^ This bacteriostatic property could be confirmed by the number of *Sd1* counted in the stool. The bacterial population decreased moderately in WOb‐treated rats compared to norfloxacin‐treated rats. Diarrheal rats developed soft stools, glary or mucus‐linked lumpy stools, and feces with a fetid odor that evidenced the presence of pus that are typical signs of infectious or “invasive” diarrhea.^30^, ^42^

In untreated diarrheic rats, destruction of the colonic mucosa and significant NO production were due to the pathogenic effects of *Shigella*. However, the protection of the colonic mucosa and the significant decrease in NO production in the colon and in the blood appear to be a direct result of treatment with the extract. The pathogenesis of *Shigella* is multifactorial and includes the production of shiga‐toxin and its ability to penetrate and destroy host tissues that largely induces an inflammatory response. *Shigella* would penetrate the intestinal epithelial barrier through M cells that cover the lymphoid follicles and reach the basolateral layer of the intestine where it can invade. *Shigella*, once in the cells, multiplies rapidly and spreads to adjacent cells.^4^ As a result of this pathogenesis, severe tissue damage in sigmoid colon and rectum, responsible for the severe dysenteric syndrome, occurs.^42^ High levels of NO in DC rats might result from the lipopolysaccharides or the enterotoxins (shiga toxin) produced by *Sd1*, which is very often implicated in the inflammation associated with diarrhea. Bacterial lipopolysaccharides or *Sd1* enterotoxins induce the expression of the inducible NO synthase gene in different inflammatory and tissue cells for the production of NO.^43^, ^44^ High bacterial load (high concentration of enterotoxins) would be responsible for the high NO level in the colon and in the blood. The extract reduced the bacterial load, causing a decrease in enterotoxins, which would lead to a decrease in NO production. O barrelieri aqueous extract contains anthocyanidins^18^ with antioxidant properties.^45^ Reduction of oxidative stress by this compound would result in decreased NO and damage to the intestinal mucosal barrier, leading to a reduction in diarrhea.^45^ Various physiological and physiopathological responses in the human body are regulated by NO molecules. It regulates blood circulation, platelet function, host defense system, and neurotransmission in the central nervous system and peripheral nerves.^43^ Blood parameters revealed low values of RBC and WBC in DC rats. Shigellosis induces anemia by loss of blood (RBC and WBC) in the feces.^46^ Nitric oxide also promotes the migration of leukocytes to the inflammatory focus.^43^ The production of IL‐1β and the recruitment of neutrophils through the intestinal epithelial layer induced by *Shigella* are responsible for the apoptosis of macrophages^4^ that could lead to a decrease of the total number of white blood cells in blood circulation.^30^ Rats treated with 50 mg/kg O barrelieri extract exhibited low levels of WBC, RBC, Ht, and Hb. This could be explained by the ineffectiveness of this dose on *Shigella* or by the high level of NO that would favor the migration of leukocytes to the infected site.^43^

O barrelieri extract was found to be bacteriostatical, inhibited bacterial growth in vivo, and reduced NO production. Furthermore, O barrelieri prevented the destruction of colonic mucosa and death in infected rats. These results attest the antishigellosis properties of O barrelieri extract. These multiple results would support the use by traditional healers, of the decoction of O barrelieri as an antidiarrheal drug. In prospect, future studies performing detailed chemical profiles of the extract will be needed to get a clear and detailed description of the major compounds of the extract and the nature of the chemicals underlying the reported antidiarrheic effect.

## CONFLICT OF INTEREST

None declared.

## AUTHOR CONTRIBUTIONS

Conceptualization: René Kamgang, Michel Archange Fokam Tagne

Formal Analysis: Michel Archange Fokam Tagne

Funding Acquisition: René Kamgang

Investigation: Michel Archange Fokam Tagne, Paul Aimé Noubissi, Gaëtan Olivier Fankem

Methodology: René Kamgang, Michel Archange Fokam Tagne

Project Administration: René Kamgang, Michel Archange Fokam Tagne

Resources: René Kamgang, Michel Archange Fokam Tagne

Supervision: René Kamgang

Validation: René Kamgang

Writing—Original Draft Preparation: Michel Archange Fokam Tagne

Writing—Review & Editing: Michel Archange Fokam Tagne, Paul Aimé Noubissi, Gaëtan Olivier Fankem, René Kamgang.
